# Successful stimulation of myocardial ganglionic plexi by Tau-20 in the absence of cardiac damage

**DOI:** 10.3389/fphys.2025.1536362

**Published:** 2025-03-28

**Authors:** Shengzhe Li, Jamie A. Kay, Danya Agha-Jaffar, Cindy S. Y. Gao, Justin Perkins, Simos Koutsoftidis, Emm Mic Drakakis, Chris D. Cantwell, Liliang Wang, Prapa Kanagaratnam, Rasheda A. Chowdhury

**Affiliations:** ^1^ Imperial College London, National Heart and Lung Institute, London, United Kingdom; ^2^ The Royal Veterinary College, London, United Kingdom; ^3^ Imperial College London, Department of Bioengineering, London, United Kingdom; ^4^ Imperial College London, Department of Aeronautics, London, United Kingdom; ^5^ Imperial College London, Department of Mechanical Engineering, London, United Kingdom

**Keywords:** atrial fibrillation, ganglionated plexi, high-frequency stimulation, Tau-20, Langendorff

## Abstract

Atrial fibrillation (AF) is a major healthcare burden worldwide. The standard invasive treatment for AF that is resistant to pharmacological intervention is a pulmonary vein isolation (PVI) procedure. Ganglionated plexus (GP) ablation can be used as an adjunctive therapy to PVIs, which together reduce the likelihood of AF recurrence. High-frequency stimulation (HFS) is a technique used to identify ectopy-triggering GP sites. However, to locate GP sites, sequential HFS must be delivered over the whole atria. Therefore, ensuring the safety of HFS delivery is integral to avoid irreversible damage from excessive pacing. We tested the Tau-20 version 2 neural simulator, a prototype of a custom-built novel electrophysiological pacing and recording system (patent reference: ASW100372P.EPP) that has the potential to guide intracardiac AF treatments. Using an *ex vivo* porcine Langendorff model that closely resembles the anatomy and physiology of a human heart, we confirmed that HFS can successfully trigger AF, suggesting that HFS-positive locations contain GP sites. Additionally, we found that HFS delivered via Tau-20 version 2 did not cause any damage to the heart. These findings are evidence that once fully optimized, the Tau-20 system could be suitable for use in clinical settings.

## 1 Introduction

Atrial fibrillation (AF) is one of the most common cardiac arrhythmias, affecting around 59.7 million people worldwide (Roth et al., 2020). Over the last two decades, the incidence of AF has increased by 31% as its risk increases with age (Lippi et al., 2021). AF leads to inefficient blood flow, which increases the risk of blood clots and, therefore, stroke occurrence (Biase et al., 2014).

Symptomatic AF is resistant to pharmacological interventions, so invasive pulmonary vein isolation (PVI) procedures (Haïssaguerre et al., 1998) have remained the cornerstone of AF treatment over the past few decades (Calkins et al., 2017). However, permanent rhythm control is not always achieved with PVI, even with improvements to both the technique and equipment. Moreover, it has been shown that the 2-year recurrence rate of AF is 5.8% and increases to 25.5% at 5 years (Shah et al., 2008).

Recent studies have shown that the intrinsic cardiac autonomic nervous system (ANS) plays a crucial role in initiating spontaneous AF (Hou et al., 2007; Schauerte et al., 2001). ANS innervation of the heart occurs through an extensive epicardial neural network of autonomic ganglia, known as the “ganglionated plexi” (GPs). The GPs include both adrenergic and cholinergic nerves and release the respective neurotransmitters. Autonomic nerve stimulation has been shown to reduce action potential duration and thereby lead to increased arrhythmia formation in the pulmonary veins (Patterson et al., 2005; Petraitiene et al., 2014). Despite their proximity, with some GP sites located near the PV ostium, it is possible that some GP sites may not be ablated by PVI alone (Po et al., 2009). Evidence suggests that targeted GP ablation in addition to PVI can improve treatment success rate and reduce AF recurrence at 12 months post-intervention (Scherlag et al., 2005; Pappone et al., 2004).

High-frequency stimulation (HFS) is a technique used to locate GP sites. Sites positive for ectopy triggering (ET) or AF on HFS stimulation are considered sites of GP due to atrial arrhythmias being triggered at a rate faster than during the local myocardial refractory period (Kim et al., 2020). An important factor to consider when sequentially mapping GP with HFS is to provide safe and effective stimulation. Stimulation does not generally lead to significant myocardial damage when applied within standard clinical parameters. However, if delivered inappropriately, factors such as stimulation intensity, location, and duration can influence its effects on myocardial tissue (Dorwarth et al., 2003; Sugrue et al., 2022). Therefore, before applying a prototype stimulator in human trials, it is necessary to test the safety of the system.

Although in the cardiac catheter laboratory pacing is primarily performed on the endocardial surface, there are alternate clinical scenarios where epicardial pacing is used and is deemed fully adequate, such as the placement of temporary pacing wires postsurgery. For ease of location visualization, *ex vivo* heart epicardial pacing is preferable (Waqanivavalagi, 2024).

A prototype of a potential clinical pacing and mapping system (Tau-20 version 2) was tested in this study using an *ex vivo* porcine model to demonstrate that GP sites can be successfully stimulated with HFS without causing any damage to the tissue. Additionally, the proportions of tissue and fat thickness were characterized to investigate the relationships between ET-GP sites and these factors.

## 2 Methods

The *in vivo* heart extraction protocol was approved by the Royal Veterinary College (RVC) Animal Welfare and Ethical Review Board. All animals were housed and transported under conditions specified in the UK *Animal Welfare Act 2006* and the *Welfare of Farm Animals (England) Regulations 2007*. Animal care, investigations, and euthanasia were performed according to the *Animals (Scientific Procedures) Act 1986*. Female Large White pigs were supplied by the RVC (Home Office project licence number PP1385023).

### 2.1 Pig preparation and heart explantation

Heart explantations were carried out at the RVC. Four healthy 4- to 5-month-old white female pigs (Large White) weighing 70–80 kg were rested for at least a week before heart extraction to allow for acclimatization. Ketamine (20 mg/kg) and midazolam (0.5 mg/kg) were injected intramuscularly (IM) for the purpose of premedication. Propofol (2–4 mg/kg) was used to induce anesthesia via a 22-gauge intravenous catheter placed in the auricular vein (Carestation 650, GE Healthcare, United Kingdom). Anesthesia was maintained using inhaled sevoflurane (SevoFlo, Zoetis, United Kingdom). Fentanyl (delivery rate: 0.2 mcg/kg/min) was applied via intravenous constant rate infusion (CRI) to achieve analgesia after an initial loading dose of 2 mcg/kg. Multiple vital signs were monitored to ensure welfare and the quality of the heart: blood pressure was measured using a catheter (Leadercath, Vygon, United Kingdom) placed in the femoral artery; body temperature, electrocardiogram, capnography, and other metrics were measured using a multiparameter monitor (Carestation 650, GE Healthcare, United Kingdom). The aim was to maintain all these within normal limits during the procedure. Heart rate was maintained at 70–120 beats per minute in sinus rhythm and the mean arterial blood pressure above 70 mmHg. Controlled mechanical ventilation was used to maintain normocapnia (35–45 mmHg), and the respiratory rate was 12–15 breaths per minute. Volume-controlled ventilation was chosen at tidal volume 6–8 mL/kg/minute, peak inspiratory pressure was set at 20 mmHg, and positive end-expiratory pressure was set at 5 mmHg for the entire procedure. Oxygen saturation was considered normal above 96%. No deviations from the normal limits were experienced.

The heart was flushed using cardioplegia solution (Sterile Concentrate for Cardioplegia Infusion, Martindale Pharmaceuticals Ltd., United Kingdom) during the procedure. An overdose of 0.7 mL/kg pentobarbital was applied to perform euthanasia at the end of heart retrieval. The procedure was similar to that used in our previous studies (Brook et al., 2020; Kim et al., 2022).

### 2.2 Explanted whole heart and Langendorff heart preparation

All hearts were retrogradely perfused via the aorta with cardioplegia solution mixed with heparin (12500 IU/L, Panpharma, France) before removal from the chest. All hearts had a minimum of 3 cm of the ascending aorta remaining after extraction to allow cannulation for the Langendorff system. The extracted hearts were stored in ice-cold cardioplegia solution for transportation. The maximum cold ischemic time was 90 min.

A custom-built Langendorff apparatus was constructed to keep the hearts alive for around 3–5 h (Brook et al., 2020). The apparatus consisted of a water heater to warm the solution to 37°C, a two-layer 5-L solution reservoir (custom supply from Radnoti Ltd.). The water-jacketed reservoir was maintained at 40°C. Heated water was pumped into the outer layer to heat up the solution, with an oxygen supply to oxygenate the physiological solution to maximal saturation. A heating coil (custom supply from Radnoti Ltd.) was located distal to the reservoir to keep the physiological solution flow to the heart at the optimal temperature of 37 ± 0.5°C. A bubble trap (custom supply from Radnoti Ltd.) was used to remove the air bubbles in the solution to prevent potential damage to the hearts, and a high flow peristaltic pump (Cole-Parmer, United Kingdom) was used to circulate the solution around the system at a constant rate (90 rpm, flow rate 500 mL/min). The physiological solution was oxygenated with Tyrode’s solution (10–3 moL/L: NaCl, 130; KCl, 4.05; MgCl_2_, 1.0; NaHCO_3_, 20; NaH_2_PO_4_, 1.0; glucose, 5.5; and CaCl_2_, 1.35; pH = 7.4).

**FIGURE 1:**
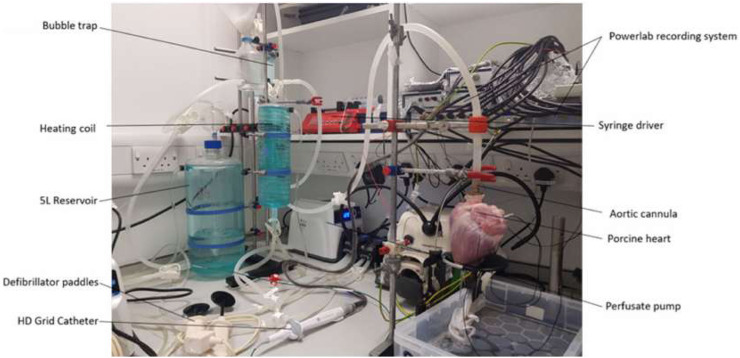
Langendorff apparatus, pump, and aortic cannula feeding into a porcine heart.

The solution chambers were connected to the aortic cannula and oxygenated Tyrode’s solution (95% oxygen/5% carbon dioxide medical gas mixture, BOC Medical, United Kingdom) was perfused (perfusion rate 500 mL/min) into the coronary arteries in a retrograde manner, thereby maintaining the metabolic, electrical, and contractile activity of the porcine hearts. The perfusate exited the coronary circulation into the right atrium and was subsequently expelled from the heart. On restarting, the heart was cardioverted using a Lifepak 20e defibrillator (Physio-Control, Inc., United States). A single lead ECG (AD-Instruments, Oxford, United Kingdom) was recorded on LabChart (AD-Instruments, Oxford, United Kingdom) to monitor the condition of the heart. During stabilization, the heart was paced from the basal region of the left ventricle with a 40-mA current using a clinical stimulator (Micropace EP Inc., United States). Left ventricular pacing was chosen during stabilization to prevent potential confounding influences of atrial pacing before HFS stimulation and electrogram (EGM) recording. The stabilization period normally lasts 20 min. The pacing was stopped once the heart returned to spontaneous beating. The HFS protocol was implemented once the heart was stabilized (sinus rhythm was consistently obtained for 15 min). At the end of the live experiments, the heart was fixed with Tyrode’s solution-buffered formalin solution and stored in a 4 C cold room. The Langendorff setup is shown in Figure 1.

### 2.3 High-frequency stimulation

**FIGURE 2:**
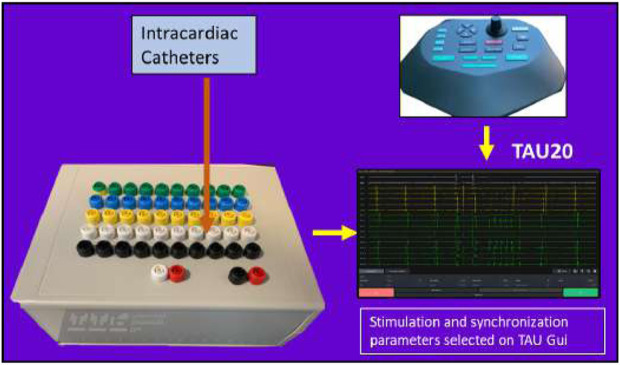
Tau-20 neural simulator system.

Tau-20 version 2, a prototype of a neural simulator, was used in the study to generate HFS and record electrograms (EGMs). Tau-20 has similar functions to the Grass stimulator, which is commonly used in clinical studies and research. It cannot be purchased or maintained in several countries due to the IEC/EN 60601-1 Third Edition Regulatory Standards. The Tau-20 system is shown in Figure 2.

Tau-20 can be linked to any mapping and ablation commercial catheter to capture EGM recordings and deliver pacing and ablation through the catheter. As HFS is widely used as an associated procedure to PVI, in addition to the functions of current commercial stimulators, Tau-20 has the capability to deliver stable HFS.

The Tau-20 system has two hardwires: a stimulator combined with a breaking box (190 mm*140 mm*70 mm) and a pentagon-shaped console (edge length: 130 mm). Tau GUI software was used. All pacing settings can be input from the GUI or the console, and it shows real-time unipolar and bipolar EGM recordings.

Tau-20 has 50 recording channels, including one ground channel and one reference channel, so it can simultaneously record from 48 electrodes. It also has two pacing channels.

A blanking amplifier has been implemented to attenuate pulse artifacts prior to filtering. The blanking switch is controlled directly by the stimulator to match the timing of stimulation. A second-order Sallen–Key high-pass filter was used to attenuate DC drift at the electrode–electrolyte interface caused by stimulation and artifacts from catheter motion. The high-pass filter cutoff frequency can be set to 0.08, 1, 30, or 60 Hz; the lowest cutoff frequency is suitable for recording lower frequency potentials such as T waves but at the expense of reduced DC offset rejection and slower transient response in the case of saturation. A first-order low-pass filter with a cutoff frequency set to 1 KHz was implemented on board to attenuate high-frequency interference. The sampling resolution was 24 bit and the sampling rate was 1,000 Hz. The stimulation output range of the Tau system was 0–80 mA (up to 14 V), and the stimulation resolution was 16 bit.

The pulse width for continuous HFS was 1 ms, and HFS was delivered to six to ten locations on the epicardial surface of the right and left atria of each heart, depending on size. HFS was delivered using a 4-mm tip ablation catheter (Abbott, United States). The atria and appendages were paced at high output (10 v) at a fixed rate (600 ms) to ensure that there was no ventricular capture. Once confirmed, HFS was delivered (pacing rate: 40 Hz; amplitude: 50 mA; duration: 5 s). This amplitude of stimulation was chosen to capture at least 1.5 times the threshold of activation and enable definitive characterization of HFS damage. The distance between each stimulation point was 15–20 mm. EGMs were recorded next to the pacing points using an Inquiry™ AFocus II™ Double Loop Catheter (Abbott, United States). A point was considered an ectopy-triggering (ET)-positive site if a positive response was obtained from the real-time EGM recording after the HFS. Positive responses include atrial ectopy, atrial tachycardia, and AF. In most of the cases, it was AF. If no arrhythmia was captured, the stimulation point was considered an ET-negative site. All the ET sites were tested at least twice to ensure reproducibility. All ET-positive and ET-negative sites were labeled with sutures to ensure they could be accurately dissected after removal from the Langendorff apparatus for further imaging and staining.

### 2.4 Histology of stimulated points

Tissue blocks (length, width: 2 mm; depth: from epicardium to endocardium) from each stimulated point and non-HFS control points were dissected from the heart once it was removed from the Langendorff apparatus, and the image of the whole heart was taken before fixing the heart with formalin solution. The tissue block was wrapped with a layer of Parafilm and another layer of aluminum foil. The wrapped block was immediately placed on dry ice for 15 min before being moved to a −80°C freezer.

The frozen tissue block was then cut into 10-µm-thick sections (around 20 sections per block) using an OTF5000 Cryostat (Bright Instruments, United Kingdom) as cross-sections from the epicardium to endocardium. The sections (two on one slide) were then adsorbed onto the adhesion slides (Avantor, United States), and the prepared slides were kept at −20°C for at least 24 h.

Hematoxylin and eosin (H&E) staining was carried out using the H-3502 Hematoxylin and Eosin Stain Kit (Vector Laboratories, United States) to test for tissue damage from HFS. The slides were removed from the freezer, allowed to dry, and fixed in ice-cold acetone (Avantor, United States) for 20 min. The slides were then stained with hematoxylin for 30 s followed by two rounds of Milli-Q water washes (first: 20 s; second: 10 s). The slides were then stained with bluing reagent for 30 s and washed with Milli-Q water for 20 s. The final step of staining was 10 s of eosin, and the slides were washed with Milli-Q water for 20 s and dehydrated with ethanol (Avantor, United States). Dehydration comprised four steps: 70% ethanol for 30 s, 90% ethanol for 30 s, and two rounds of 100% ethanol for 30 s. After dehydration, the samples were cleared three times (5 min each) using xylene (Honeywell, United States) and coverslips were applied with DPX mounting media (Sigma, United States).

**FIGURE 3:**
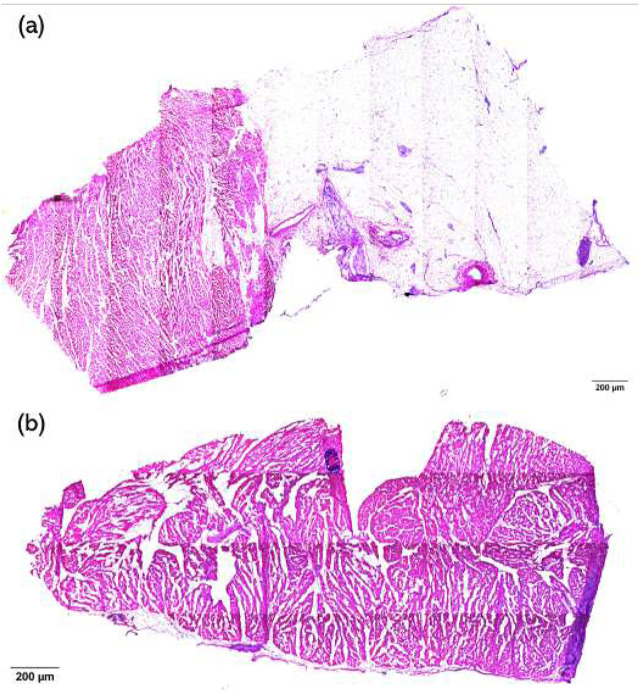
**(a)** Example of tissue with level 2 damage; **(b)** example of tissue with level 3 damage.

H&E staining of the samples was used to visualize the damaged condition of the tissue, and a semi-quantitative scoring system was used to measure the level of damage to the tissue. The damage level score is shown in Table1. Images were scored by eye, where a score of 1 was given for images with no damage and a score of 5 was given when necrosis extended to the endocardial surface. If any sample exhibited multiple damage levels, the highest damage level was recorded. Examples of level 2 and 3 damage are shown in Figure 3.

**TABLE 1:** Damage level scores and their description. Semi-quantitative scoring system was used to assess the damage level. Score of 1 is given when there is no damage. Score of 5 is given when the necrosis extends to the endocardial surface.

Damage level	Damage description
1	No damage
2	Slight damage in the epicardial area
3	Slight damage in the entire area
4	Necrosis on the epicardial surface
5	Necrosis on the endocardial surface

The scores are highest from two individual observers with expertise in histological H&E staining and evaluation. If the scores between observers were not in consensus, a third scorer was asked to score it again and provide score consensus. All images were blinded before scoring.

### 2.5 Imaging and analysis

Prior to dissection of the atria, the whole heart was imaged at 360° using a Nikon camera from the top, middle, and bottom, and these images were used to make a 3D model of the heart using ReCap Pro 2024 (Autodesk, United States).

The stained slides were imaged using a Zeiss Axio Observer inverted microscope controlled by ZEN Pro software (Zeiss, Germany) at Imperial College, London. The objective used was ×10, and brightfield images were taken to examine the damage. The epicardial, myocardial, and connective tissue layers of each sample were determined by manually selecting the distinct structures by histological examination, and their area was measured automatically using the software.

Image analysis was performed using ImageJ (FIJI) software. The thickness of the samples was recorded by measuring the distance between the epicardium and the endocardium. The length of the fat layer was also measured to verify whether there was any difference in fat and myocardial thicknesses between ET-positive and ET-negative sites. A semi-quantification system was applied to verify the damage level of the samples.

All images were blinded for analysis.

### 2.6 Statistical analysis

The study is reported in accordance with the ARRIVE guidelines. GraphPad 10 (Prism, United States) was used for all statistical testing. One-way ANOVA was performed to compare the data collected from three different groups. A Tukey’s multiple comparison test was carried out as a post hoc correction. The threshold for statistical significance was set to *p* < 0.05.

## 3 Results

**FIGURE 4:**
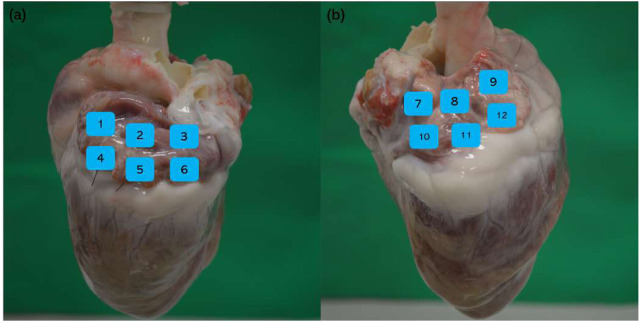
Example of HFS delivered points and non-HFS control points. **(a)** Left atrium: positions 1 and 4 are non-HFS control points; positions 2, 3, 5, and 6 are HFS delivered points; **(b)** right atrium: positions 7 and 10 are non-HFS control points; positions 8, 9, 11, and 12 are HFS delivered points.

**FIGURE 5:**
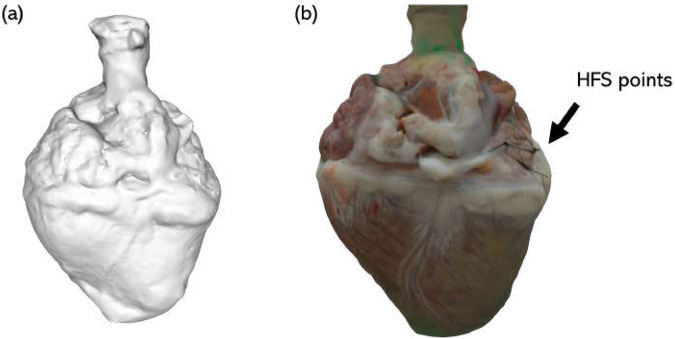
Example 3D image of a porcine heart. **(a)** Grayscale composite model; **(b)** 3D reconstructed model with real color. Sites of HFS delivery are demarcated by sutures (indicated by black arrow).

Four porcine hearts were tested. HFS was delivered to 36 points of the atria to cover the majority of the left and right atria without overlap. An example of the locations is shown in Figure 4. Any sustained AF longer than 10 s (normally longer than 30 s) after cessation of HFS was considered positive in this study. Four sites in two hearts demonstrated ET-positive locations. The longest AF obtained from the negative sites after HFS at the negative sites was 3 s. Twelve samples distant from the area of stimulation ( > 2mm) were also saved for histology as negative controls. Within the four ET-positive sites, two were from the left atrium and two were from the right atrium. Detailed information of samples from each heart is shown in Table 2. An example of the 3D model image of the hearts for electrode localization is shown in Figure 5.

**TABLE 2:** Numbers of ET-positive, ET-negative, and control sites per heart.

Heart number	Left			Right		
	ET-positive site	ET-negative site	Control site	ET-positive site	ET-negative site	Control site
1	1	4	1	0	5	2
2	1	4	2	1	4	1
3	0	5	1	0	5	2
4	0	5	1	1	4	2

### 3.1 AF triggered by HFS

**FIGURE 6:**
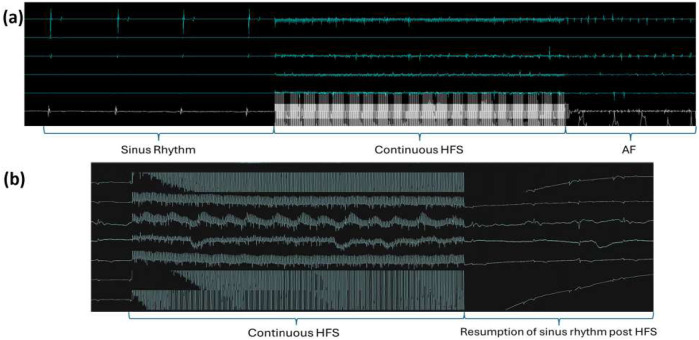
Examples of EGM recordings. **(a)** Example of an HFS-tested site triggering reproducible ectopy/AF. Sinus rhythm was captured before the delivery of HFS and all the electrical activities of the heart were obscured by the HFS signal during its delivery. AF was captured after HFS, and the same site was tested 2–3 times, consistently triggering AF in all instances. **(b)** Example of a negative site. As with the positive sites, sinus rhythm was captured before and after HFS, and only the HFS signal was recorded during the stimulation.

Sinus rhythm and HFS were recorded from all locations before and during the delivery of HFS. From the 32 negative sites, the EGM reverted to sinus rhythm immediately after the HFS; from the four ET-positive sites, AF was captured after the HFS. Examples are shown in Figure 6.

### 3.2 Damage level of the samples

**FIGURE 7:**
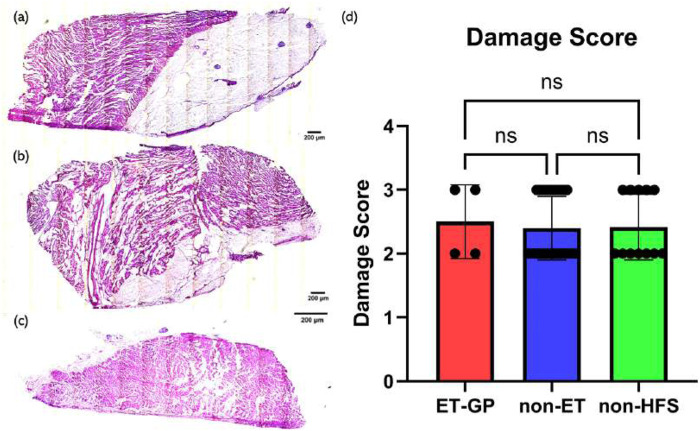
H&E staining examples and the damage score with relation to HFS. **(a)** Example of H&E staining of a sample from an ET-positive site. **(b)** Example of H&E staining of a sample from an ET-negative site. **(c)** Example of H&E staining of a sample from a non-HFS site. **(d)** Damage score with relation to HFS. Samples from 4 ET-positive sites, 32 ET-negative sites, and 12 non-HFS sites were analysed. *P*-value = 0.9337. At least two samples were scored from each tissue, and the highest score was chosen to represent the damage level of the tissue. One-way ANOVA statistical analysis was used to assess significance. Mean values are represented by each bar. Dots represent the individual value of each heart. Error bars represent the standard deviation. Statistical analysis results are shown on the top bracket, where ns means not significant if the *p*-value ≥ 0.05.

To investigate whether damage was caused by the HFS delivered from the Tau-20 neural simulator, H&E staining was carried out on the samples from all the tissues (4 ET-positive sites, 32 ET-negative sites, and 12 sites without HFS delivery) procured after the live protocols. At least two 10-um tissue sections from each site were stained and the damage levels were quantified. There was no significant difference between the damage levels of ET-positive sites (2.5 ± 0.58), ET-negative sites (2.4 ± 0.50), and no HFS sites (2.42 ± 0.51), with a *p*-value of 0.93 (between GP-positive and non-GP), 0.93 (between non-GP and non-HFS), and 0.94 (between GP-positive and non-HFS), respectively. Examples of the H&E staining and group data are shown in Figure 7.

### 3.3 Comparison of myocardial thickness and fat thickness between ET-positive, ET-negative, and control sites

**FIGURE 8:**
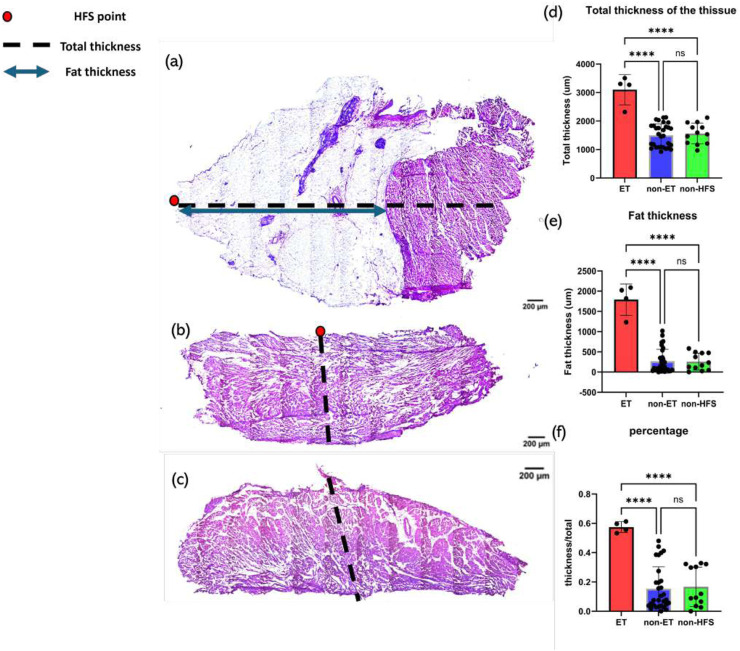
Examples of thickness measurements and thickness analysis. The red dot depicts the HFS-positive site, the black dashed line represents total thickness, and the blue line represents fat thickness. **(a)** Example of thickness measurements of a sample from an ET-positive site. **(b)** Example of thickness measurements of a sample from an ET-negative site. **(c)** Example of thickness measurements of a sample from a non-HFS site. **(d)** Tissue response to HFS as a function of total tissue thickness. *P*-value < 0.0001. **(e)** Tissue response to HFS as a function of fat thickness of the tissue. *P-*value < 0.0001; **(f)** Tissue response to HFS as a function of the ratio of fat thickness to total thickness of the tissue. *P*-value < 0.0001. Samples from 4 ET-positive sites, 32 ET-negative sites, and 12 non-HFS sites were analyzed. At least two samples were measured from each tissue, and the mean values were calculated to represent the thicknesses. One-way ANOVA was used to assess statistical significance. Result was considered significant if the *p*-value ≤ 0.05. Number of asterisks represents different significance levels: * = *p*-value ≤ 0.05; ** = *p*-value ≤ 0.01; *** = *p*-value ≤ 0.001; **** = *p*-value ≤ 0.0001.

To explore whether there is a correlation between myocardial thickness and fat thickness in ET-positive, ET-negative, and control sites, total myocardial thickness, fat thickness, and their proportion in all tissues (4 ET-positive sites, 32 ET-negative sites, and 12 sites without HFS delivery) were compared. At least two sections from each site were measured. The mean thickness value measured from samples from the same tissue was calculated to represent the thickness of the whole tissue and the thickness of fat. ET-positive sites (total thickness: 3,098.8 um ± 530.5 um; fat thickness: 1790.88 um ± 388.9 um; mean percentage: 0.57 ± 0.03) demonstrated significantly increased wall thickness, fat thickness, and ratio compared to ET-negative (total thickness: 1500.23 um ± 404.6 um; fat thickness: 267.65 um ± 296.8 um; mean percentage: 0.15 ± 0.15) and non-HFS (total thickness: 1563.54 um ± 366.2 um; fat thickness: 258.38 um ± 210.4 um; mean percentage: 0.17 ± 0.13) sites. No significant differences were observed between ET-negative and non-HFS sites. For total thickness, the *p*-value between ET-positive and ET-negative sites was ≤0.0001, the *p*-value between ET-positive and non-HFS sites was ≤0.0001, and the *p*-value between ET-negative and non-HFS sites was 0.89. For fat thickness, the *p*-value between ET-positive and ET-negative sites was ≤0.0001, the *p*-value between ET-positive and non-HFS sites was ≤0.0001, and the *p*-value between ET-negative and non-HFS sites was 0.99. Examples of the measurement and the results are shown in Figure 8.

## 4 Discussion

We have demonstrated that HFS delivered by Tau-20 version 2 sufficiently triggered AF and localized GP sites without damaging the tissue. However, some damage was observed in all tissue samples irrespective of HFS. Therefore, these injuries were likely caused by Langendorff perfusion. As the heart was placed on the Langendorff system for several hours, a slightly higher perfusion fluid pressure than that in *in vivo* conditions may have led to myocardial edema and damage—injuries that have been illustrated in several studies (Bell et al., 2011; Walters et al., 1992; Qiu and Hearse, 1992). The perfusion pressure and solution temperature were held constant during the experiment but were higher than that in *in vivo* conditions to ensure adequate oxygen delivery, which may have led to damage after hours of experimentation. However, the comparison between the negative control (non-HFS sites: no significant difference) within the same hearts demonstrated that the damage was not likely due to the HFS. One limitation is that the low-level damage caused by HFS can be hidden under the water damage.

This study also illustrates that there is a clear link between tissue and fat thickness with ET-positive locations. We have previously characterized the neuronal composition of ET-positive sites (Kim et al., 2020). In this study, we demonstrated that ET-positive sites are located in areas of greater wall thickness, fat thickness, and fat/tissue thickness ratio than in ET-negative sites. This suggests that GPs are more likely to exist in areas with higher fat thickness, while areas with less fat do not have sufficient space to incorporate GP sites. But the mechanism by which GP sites co-locate with fat is not clear yet and may be of interest for future studies. However, a link between increased epicardial fat, atrial remodeling, and increased AF burden has been identified (Conte et al., 2022). Therefore, it is possible that this general mechanism may involve an increase in GP density and/or trigger co-localization with fat.

Currently, the most common method of locating sites in clinical practice is the delivery of a long period of HFS across the atria, which is time-intensive and also risks triggering fibrillation if the HFS is not delivered properly (Po et al., 2009; Choi et al., 2017; Coyle et al., 2023). When characterizing GP sites around the atrioventricular junction, HFS can be mistakenly performed on the ventricle, which may trigger VF. Therefore, these novel correlations with wall thickness could provide another potential GP-locating method for treatments and therefore reduce the risk. If wall thickness is assessed prior to HFS protocols through noninvasive imaging techniques, such as cardiac magnetic resonance imaging, HFS delivery could be minimized to only the requisite areas. However, since only four porcine hearts were tested, further studies are required to establish the generalizability of this finding and also verify if such a trend can be found in humans. Current clinical GP research is not focused on such a relationship, so to date there is no evidence showing that this assumption can be applied to human hearts. However, using noninvasive imaging to guide HFS delivery is promising and could be a potential future research direction.

In this experimental setting, pacing was undertaken from the epicardial surface for ease of location visualization. Clinically, in the postsurgical setting, the epicardial surface of either the ventricle or atrium is deemed a suitable location for placing temporary pacing wires. Due to the thinness of the atrial wall, the assumption was made that activation was through the full wall thickness (Waqanivavalagi (2024).

In addition, only four pig hearts were used in this study. Although multiple sites per heart were tested, data from more hearts and additional species could further enhance reliability and applicability.

In conclusion, Tau-20 shows promise as a novel platform for HFS to identify GP sites, and co-localization with areas of fat needs further investigation.

## Data Availability

The raw data supporting the conclusions of this article will be made available by the authors, without undue reservation.
